# Acute respiratory failure as presentation of late-onset Pompe disease complicating the diagnostic process as a labyrinth: a case report

**DOI:** 10.1186/s40248-018-0145-4

**Published:** 2018-09-01

**Authors:** Francesco Menzella, Luca Codeluppi, Mirco Lusuardi, Carla Galeone, Franco Valzania, Nicola Facciolongo

**Affiliations:** 1 Department of Medical Specialties, Pneumology Unit, Arcispedale Santa Maria Nuova, Azienda USL di Reggio Emilia- IRCCS, Via Amendola 2, 42122 Reggio Emilia, Italy; 2 Neuromotor & Rehabilitation Department, Neurology Unit, Arcispedale Santa Maria Nuova, Azienda USL di Reggio Emilia-IRCCS, Reggio Emilia, Italy; 3 Unit of Respiratory Rehabilitation, Azienda USL di Reggio Emilia, S. Sebastiano Hospital, Correggio, Italy

**Keywords:** Respiratory failure, Glycogen, Enzyme, Muscle biopsy, Ventilation

## Abstract

**Background:**

Acute respiratory failure can be triggered by several causes, either of pulmonary or extra-pulmonary origin. Pompe disease, or type II glycogen storage disease, is a serious and often fatal disorder, due to a pathological accumulation of glycogen caused by a defective activiy of acid α-glucosidase (acid maltase), a lysosomal enzyme involved in glycogen degradation. The prevalence of the disease is estimated between 1 in 40,000 to 1 in 300,000 subjects.

**Case presentation:**

This case report describes a difficult diagnosis of late-onset Pompe disease (LOPD) in a 52 year old Caucasian woman with acute respiratory failure requiring orotracheal intubation and subsequent tracheostomy for long-term mechanical ventilation 24 h/day. Despite a complex diagnostic process including several blood tests, bronchoscopy with BAL, chest CT, brain NMR, electromyographies, only a muscle biopsy allowed to reach the correct diagnosis.

**Discussion:**

The most frequent presentation of myopathies, including LOPD, is proximal limb muscle weakness. Respiratory related symptoms (dyspnea on effort, reduced physical capacity, recurrent infections, etc.) and respiratory failure are often evident in the later stages of the diseases, but they have been rarely described as the onset symptoms in LOPD. In our case, a third stage LOPD, the cooperation between pulmonologists and neurologists was crucial in reaching a correct diagnosis despite a very complex clinical scenario due to different confounding co-morbidities as potential causes of respiratory failure and an atypical presentation. In this patient, enzyme replacement therapy with infusion of alglucosidase alfa was associated with progressive reduction of ventilatory support to night hours, and recovery of autonomous walking.

## Background

Respiratory failure can be triggered by several causes, either of pulmonary or extra-pulmonary origin, including progressive neuro-muscular disorders [1].

Pompe disease (PD), also known as type II glycogen storage disease or lysosomal acid α-glucosidase (acid maltase) deficiency, is a serious and often fatal condition, due to an autosomal recessive genetic alteration of glycogen metabolism. The predominant clinical manifestation is a neuromuscular disease with varying degrees of progression [2, 3]. PD can appear at different ages with varying phenotypes and involvement of different organs. The prevalence may vary among ethnic groups and geographical areas and is estimated to occur in 1:40,000 to 1:300,000 subjects [3].The clinical presentation and the severity of the disease depends on the residual activity of the enzyme; absence of detectable enzymatic activity is typical of the most severe forms. In symptomatic patients, diagnosis of PD is based on the low acid α-glucosidase activity in skin fibroblasts or blood samples [4].

Patients with late-onset Pompe disease (LOPD) are classified as pre-symptomatic (first stage), symptomatic (second stage) and severe (third stage), according to the degree of muscle involvement, respiratory function deterioration, ventilator dependence and need for tracheostomy [5].

Muscle biopsy can be performed to confirm the presence of a myopathy and to determine the enzyme activity. The biopsy site can influence the diagnostic yield due to the variability of glycogen content among the different muscle segments. Diagnosis is confirmed by the genetic profile [6].

In this report we describe a case of LOPD presenting as acute hypercapnic respiratory failure with carbonarcotic coma and epilepsy. While mechanical ventilation allowed to stabilize the patient, only after a difficult diagnostic process it was possible to understand the whole clinical scenario and provide an appropriate targeted therapy with specific enzyme replacement.

## Case presentation

On July 2017, a 52 year old Caucasian woman was admitted to our Emergency Department with acute respiratory failure. She had complained in the previous days of drowsiness, apathy and headache. Arterial blood gas determination showed the following values: pH 7.48, pCO_2_ 92.0 mmHg, pO_2_ 58.5 mmHg, B.E. (B) 16 mmol /L, HCO_3_ 42.6 mmol/L, SO_2_ 91.3%. Significant data at clinical history were a former smoking habit and quadrantectomy for breast carcinoma in 2012. In the latest two years she had been followed by a nutritionist for unintentional weight loss. For a long-term history of headache, 3 months before she had undergone to a brain Nuclear Magnetic Resonance (NMR) found to be normal. Due to depressive symptoms in the latest 12 months, she had been prescribed by a psychiatrist citalopram and then duloxetine without benefits.

At hospital admission chest X-ray did not show significant alterations. An electromyographic study was performed, including nerve conduction evaluation, repetitive nerve stimulation, and concentric needle examination (this latter of limited reliability for the effect of sedation on patient’s cooperation). Commonly tested nerves and muscles were explored with normal findings.

The whole clinical scenario was interpreted as an exacerbation of chronic ventilatory failure not related to a neurological cause. Actually, the presence of long-standing symptoms such as headache, tremor, drowsiness, psychic alterations were attributed to chronic hypercarbia caused by a broncho-pulmonary disorder.

In the following days, a transient clinical improvement allowed to extubate the patient. Distress of accessory muscles with rapid recurrence of respiratory failure required a prompt re-intubation. A percutaneous tracheotomy was performed with the placement of a cuffed non-fenestrated cannula (Tracoe 301–06, internal diameter 6.0 mm). The patient was ventilated in Pressure Support Ventilation (PSV) mode. A chest CT showed parenchymal consolidation areas in the right upper lobe and bilateral pleural effusion, with no evidence of diaphragm elevation. A bronchoscopy with bronchoalveolar lavage (BAL) ruled out significant alterations in the tracheobronchial tree, after removal of dense mucous exudate. Blood tests showed anemia (Haemoglobin 8.2 g/dL), absence of leukocytosis and normal values of creatine kinase (CK) (43 U/L).

The following week, a second electromyographic study confirmed normal data at limb examination. A diaphragm nerve conduction study revealed pathological low amplitude of both right and left responses as the only isolated neurophysiological alteration. The interpretation of the latter data was uncertain but a diagnostic work-up searching for primary neurological causes was scheduled. Blood CK, brain and cervical spine MRI, contrast-enhanced brachial plexus CT scan and cerebrospinal fluid examination were all found to be normal.

Following 10 days in the Intensive Care Unit, the patient was admitted to the Respiratory Intensive Care Unit (RICU) of our Pneumological ward. After a long period on continuous mechanical ventilation the patient showed a progressive clinical improvement allowing a gradual weaning. Unfortunately, a sharp increase in CO_2_ values (> 100 mmHg) was associated with the appearance of nonconvulsive status epilepticus. The patient was treated with intravenous phenytoin, with improvement in symptoms and regression of electroencephalography abnormalities (EEG). A switch to volumetric mode ventilation accelerated a good compensation of respiratory exchanges, with a progressive decrease of CO_2_ values and regression of epileptiform abnormalities on EEG. Recurrent respiratory infections sustained by various bacteria (Stenotrophomonas Maltophilia, Morganella Morganii, *Enterobacter cloacae*), required repeated cycles of targeted antibiotic therapy with piperacillin/tazobactam, cotrimoxazole, ceftriaxone, levofloxacin, meropenem.

In the following weeks the patient reached a complete recovery of awareness and collaboration, permitting to perform a quantitative electromyography testing (QEMG), which showed myopathic changes in few proximal and axial muscles. A diffused myopathy with prevalent clinical involvement of the axial and respiratory muscles was therefore hypothesized as the origin of a condition leading to respiratory muscle exhaustion. The autoantibody research was also performed to rule out neuromuscular junction diseases and autoimmune neuropathies.

A muscle biopsy was performed by incision on the left brachial biceps. The analysis confirmed myopathic changes with glycogen storage vacuoles and atrophy of type 1 and 2 fibers; enzimatic testing disclosed markedly reduced acid α-glucosidase activity in muscle tissue. A molecular analysis of the Glucosidase Alpha Acid (GAA) gene was also performed by examining DNA from peripheral blood leukocytes using new generation sequencing with Illumina MiSeq and Agilent Haloplex HS kit. The regions analyzed were the coding region and intron-exon junctions. The pathogenetic mutations were identified in heterozygous c.-32-13 T > G and c.1551 + 1G > C. Therefore, the final diagnostic outcome was “Glycogen Storage Dysease type 2” also known as LOPD.

At the end of August, enzyme replacement therapy (ERT) was started with infusion of recombinant human alglucosidase alpha (rh-GAA, Myozyme®; Genzyme Corporation, Cambridge, MA, USA), 20 mg/kg of body weight (900 mg), administered every 2 weeks.

The patient was then transferred to the Unit of Respiratory Rehabilitation and in the following months there was a progressive clinical improvement with gradual reduction of ventilatory support to night hours only, and recovery of autonomous walking. Evaluation of the diaphragmatic function was not performed since at that point it would have not contributed to modify the clinical management. A monitoring at follow up did not show any development of anti-rhGAA antibodies, without any variation of the response to the replacement treatment.

## Discussion

The most frequent presentation of myopathies, including LOPD, is proximal limb muscle weakness. Respiratory related symptoms (dyspnea on effort, reduced physical capacity, recurrent infections, etc.) and respiratory failure are often evident in the later stages of the diseases, but they have been rarely described as the onset symptoms in LOPD [7, 8].

The involvement of the thoracic muscles and of the diaphragm is the central point of the functional respiratory decline starting with reduction of vital capacity, hypoventilation during sleep [9] progressing to diurnal hypoxemia (PO_2_ < 60 mmHg) and hypercapnia (CO_2_ > 45 mmHg), cor pulmonale and finally death [9, 10]. Muscle weakness also causes ineffective cough and frequent respiratory infections that heavily contribute to functional decline. Other events which may be present are loss of balance, muscle pain and/or fatigue, contractures, ptosis, difficulty in chewing and swallowing, raised CK blood levels. These elements were absent in our patient, making the diagnosis particularly difficult.

Laboratory methods to diagnose Pompe disease are mainly represented by the analysis of the activity of the GAA enzyme and the search for mutations of the GAA gene [11]. The method usually used for the initial diagnosis is the enzyme assay, performed on fibroblasts of cultured skin or on muscle biopsies, that require invasive sampling procedures in both cases. More recently, minimally invasive assays have been developed on blood cells and dried blood spots (DBS). Unfortunately, unlike the GAA tests on fibroblasts, a clear correlation between GAA activity in DBS and clinical phenotype has not been established [11]. In our patient, muscle biopsy permitted to obtain reliable results and to perform a genetic analysis with a final definite diagnosis.

There are mainly two options to support breathing in these patients, i.e. non-invasive mechanical ventilation (NIV) and assisted cough. Replacement therapy with alglucosidase alfa (Myozyme®, Genzyme), a recombinant lysosomal glycogen-cleaving enzyme, is at present the only specific pharmacological approach for LOPD. A recent systematic literature review evaluated 22 publications and 438 patients [12]. Treatment with alglucosidase alfa had a mortality rate nearly five-fold lower compared to untreated patients. Replacement therapy was associated with a reduction in forced vital capacity (FVC) decline, an improvement in 6-min-walk test and ambulation within the first few months as well. These data confirm that early starting ERT allows the best outcomes. A recent study on 16 patients (EMBASSY study) demonstrated a significant reduction in lysosomal glycogen after 6 months of ERT, confirming the stabilization of the disease in ERT-naïve LOPD patients [13]. Formation of anti-rhGAA antibodies is a well known side effect of ERT. Compared to Gaucher and Fabry disorders the experience in Pompe disease is poor, but currently available data indicate that several PD patients on ERT develop anti-rhGAA [14, 15]. It is unknown whether sustained antibody titers occur in adults with PD, and whether high titers can modify the response to treatment. A retrospective study on sixty patients with LOPD showed that six cases (10%) developed anti-rhGAA antibodies [16]. Howewer, only three patients showed a clinical decline in terms of pulmonary function, quality of life, and motor function testing.

The management of patients with LOPD requires a multidisciplinary approach with a specific role for neurologists and pulmonologists. The pulmonologist must be particularly expert on the management of respiratory failure in both chronic and acute-on-chronic settings considering the need of mechanical ventilation for vital support in the long term.

In our case, a third stage LOPD, the cooperation between pulmonologists and neurologists was crucial in reaching a correct diagnosis despite a very complex clinical scenario due to different confounding co-morbidities as potential causes of respiratory failure and an atypical presentation. The muscle biopsy was fundamental and the rapid initiation of ERT with a proper ventilatory support were crucial for the patient’s prognosis, which rapidly improved in the following months. The pulmonologists must be aware of this rare disease, since treatment options available at present can modify significantly an otherwise unfavourable prognosis.

## Abbreviations

Anti-rhGAA: Antibodies against alglucosidase alpha
BAL: Bronchoalveolar lavage
CK: Creatine kinase
CT: Computed tomography
DBS: Dried blood spots
EEG: Electroencephalography
ERT: Enzyme replacement therapy
GAA: Glucosidase Alpha Acid
LOPD: Late-onset Pompe disease
NIV: Non-invasive mechanical ventilation
PD: Pompe disease
PSV: Pressure Support Ventilation
QEMG: Quantitative electromyography testing
rh-GAA: Recombinant human alglucosidase alpha
RICU: Respiratory Intensive Care Unit
